# Patient Self-Assessment and Acoustic Voice Analysis in Screening of Postoperative Vocal Fold Paresis and Paralysis

**DOI:** 10.1177/14574969211007036

**Published:** 2021-04-12

**Authors:** Maria Heikkinen, Elina Penttilä, Mari Qvarnström, Kimmo Mäkinen, Heikki Löppönen, Jussi M Kärkkäinen

**Affiliations:** Department of Otorhinolaryngology—Head & Neck Surgery, Kuopio University Hospital, PL 100, 70029 Kuopio, Finland Institute of Clinical Medicine, University of Eastern, Kuopio, Finland; Department of Otorhinolaryngology—Head & Neck Surgery, Kuopio University Hospital, Kuopio, Finland Institute of Clinical Medicine, University of Eastern, Kuopio, Finland; Department of Phoniatrics, Kuopio University Hospital, Kuopio, Finland; Institute of Clinical Medicine, University of Eastern, Kuopio, Finland Heart Center, Kuopio University Hospital, Kuopio, Finland; Department of Otorhinolaryngology—Head & Neck Surgery, Kuopio University Hospital, Kuopio, Finland Institute of Clinical Medicine, University of Eastern, Kuopio, Finland

**Keywords:** Thyroid surgery, parathyroid surgery, vocal fold paresis, vocal cord paresis, recurrent laryngeal nerve

## Abstract

**Background and objective:**

The aim of this study was to evaluate the utility of two items in vocal fold paresis and paralysis screening after thyroid and parathyroid surgery: patient self-assessment of voice using the Voice Handicap Index and computer-based acoustic voice analysis using the Multi-Dimensional Voice Program.

**Methods:**

This was a prospective study of 181 patients who underwent thyroid or parathyroid surgery over a 1-year study period (2017). Preoperatively, all patients underwent laryngoscopic vocal fold inspection and acoustic voice analysis, and they completed the Voice Handicap Index questionnaire. Postoperatively, all patients underwent laryngoscopy prior to hospital discharge; 2 weeks after the surgery, they completed the Voice Handicap Index questionnaire a second time. Two weeks postoperatively, patients with vocal fold paresis or paralysis and 20 randomly selected controls without vocal fold paresis or paralysis underwent a follow-up acoustic voice analysis.

**Results:**

Fourteen patients had a new postoperative vocal fold paresis or paralysis. Postoperatively, the total Voice Handicap Index score was significantly higher (p = 0.040) and the change between preoperative and postoperative scores was greater (p = 0.028) in vocal fold paresis or paralysis patients. A total postoperative Voice Handicap Index score > 30 had 55% sensitivity, and 90% specificity, for vocal fold paresis or paralysis. In the postoperative Multi-Dimensional Voice Program analysis, vocal fold paresis or paralysis patients had significantly more jitter (p = 0.044). Postoperative jitter > 1.33 corresponded to 55% sensitivity, and 95% specificity, for vocal fold paresis or paralysis.

**Conclusions:**

In identifying postoperative vocal fold paresis or paralysis, patient self-assessment and jitter in acoustic voice analysis have high specificity but poor sensitivity. Without routine laryngoscopy, approximately half of the patients with postoperative vocal fold paresis or paralysis could be overlooked. However, if the patient has no complaints of voice disturbance 2 weeks after thyroid or parathyroid surgery, the likelihood of vocal fold paresis or paralysis is low.

## Introduction

Vocal fold paresis or paralysis (VFP) caused by recurrent laryngeal nerve damage is a major complication of thyroid and parathyroid surgery, and it may inflict a lifelong handicap. VFP incidence may be up to 14%,^1–4^ averaging at 5% in most institutions.^1,3–10^ To detect a possible VFP after surgery, laryngoscopy is the gold standard diagnostic method. However, routine pre- and postoperative laryngoscopic examinations performed on all patients undergoing surgery of the neck are time-consuming and may not be cost-effective. It requires skills to rule out VFP and often special equipment such as a fiberscope. Since VFP is rather uncommon, the screening of all patients undergoing surgery results in only a few diagnoses. Although routine laryngoscopy is recommendable for early detection of postoperative VFP and for the quality control of thyroid and parathyroid surgery, it is tempting to discharge patients who have no complaints after surgery without performing laryngoscopy. This has become a topical issue especially during the 2020 global pandemic when all unnecessary close contacts with patients should be avoided.

After thyroid and parathyroid surgery, VFP is the main reason for further voice complications. However, transient laryngeal trauma caused by endotracheal intubation may also trigger voice changes, which usually disappear in 2 weeks.^11,12^ Postoperative VFP can be detected, for instance, in two ways. For subjective self-assessment of voice, patients may use the Voice Handicap Index (VHI) questionnaire.^13^ For objective acoustic voice analysis, the Multi-Dimensional Voice Program (MDVP) is currently the most commonly used and cited software.^14^ Previous studies have demonstrated that patients with iatrogenic VFP have higher VHI scores postoperatively compared to patients without VFP.^15–17^ However, to our knowledge, no previous studies have investigated VHI as a screening method for VFP after thyroid or parathyroid surgery. The aim of this prospective study was to evaluate the utility of patient self-assessment using the VHI questionnaire and acoustic voice analysis using the MDVP in VFP screening 2 weeks after thyroid or parathyroid surgery.

## Materials and Methods

All patients signed a consent form, and the study had the approval of the institutional review board. All consecutive patients undergoing thyroid or parathyroid surgery over a 1-year study period (2017) in a single tertiary hospital were considered for recruitment. Preoperatively, all patients underwent laryngoscopic vocal fold inspection as well as acoustic voice analysis using the MDVP and provided self-assessment of voice quality via the VHI questionnaire. Postoperatively, all patients underwent laryngoscopy prior to hospital discharge; 2 weeks after the surgery, they completed the VHI questionnaire a second time. Then patients with postoperative VFP and 20 randomly selected controls from the study population who had no postoperative VFP provided a second voice recording for MDVP analysis.

Preoperatively, the vocal fold function was evaluated by an otolaryngologist, who primarily performed indirect laryngoscopy; when the visibility was inadequate or suboptimal, fiberoptic laryngoscopy was used. VFP was diagnosed if vocal fold hypomobility or immobility was observed. Thereafter, while patients phonated a vowel at a comfortable frequency, a trained nurse recorded 5-s voice samples. These samples were recorded with an iOS app called OperaVox (On PErson RApid VOice eXaminer, Oxford Research Wave Ltd, UK), which was installed on an iPad air 2 (Apple Inc., Cupertino, CA, USA). The device has an internal microphone; this device as a recording system is compatible with the direct digitation method.^18^ The iPad was placed in a tablet holder, and the lips-to-device distance was 30 cm. For the voice samples, patients were in standing position unless their physical condition prevented it. Voice samples were saved in WAV files and transferred to an MDVP workstation at a different location, for logistic reasons. From each recording, 3 s of the highest quality were analyzed using the MDVP software. The acoustic parameters produced include fundamental frequency (F0), cycle-to-cycle frequency variation (jitter), amplitude variation (shimmer, shimmer dB), and the amount of additive noise in the voice signal (noise-to-harmonic ratio, NHR).^19^

Preoperatively, patients completed the VHI questionnaire at the Department of Otolaryngology. Two weeks after the surgery, patients filled out and mailed back the VHI questionnaire, a second time. The VHI questionnaire comprises 30 questions with three subscales: functional (F-VHI), physical (P-VHI), and emotional (E-VHI). Each one of these has 10 specific statements scored on a 5-point progressive numeric scale from 0 (never) to 4 (always). A partial scoring (0–40 points) for each one of the three subscales and one total score (0–120 points) is obtained.^20^ A total score between 0 and 30 reflects minimal handicap due to a voice problem; 31–60 is moderate and 61–120 is severe.^21^

Two weeks after surgery, patients with postoperative VFP and 20 randomly selected control patients underwent a second voice recording, which was performed by a speech and language therapist at the Department of Phoniatrics. The voice samples were simultaneously recorded using the iPad system and a condenser microphone compatible with MDVP software, to validate the voice recording method used in this study. The recording and analyzing of these samples were identical to the preoperative process. In addition, voice samples recorded using both these methods were studied for correlations of each of the recorded parameters.

### Statistical analysis

All statistical analyses were performed using SPSS Statistics 25.0 (IBM Corp, Armonk, NY). The parameters were tested for normality using Shapiro–Wilk test. Continuous variables are reported as mean ± standard deviation or median with interquartile range (IQR) for non-normally distributed variables. Group differences among continuous variables were tested using the Mann–Whitney U test and the Chi-square test was used for categorical data. Pearson correlation coefficient test was used to analyze the correlations between VHI and MDVP parameters. A 0.1–0.3 value represented mild correlation; 0.3–0.5 was moderate, and > 0.5 was strong. To identify the critical values at which different variables were associated with patient having VFP, Youden indexes and receiver operating characteristic (ROC) curves were generated. A Youden index indicates the performance of a test at a given cut-off value (Youden index = sensitivity + specificity − 1).

## Results

Altogether 213 consecutive patients were planned for thyroid or parathyroid surgery during the study period. Of these, 22 patients were ineligible for the study, and 10 patients were excluded after recruitment (Supplemental material; Study Flow Chart). Finally, 181 patients were included in this study (mean age 58 ± 15 years, 87% female). The indications for surgery were goiter in 71 (39%), suspicion of malignancy in 39 (22%), malignant tumor in 6 (3%), hyperthyroidism in 25 (14%), and hyperparathyroidism in 40 (22%) patients. The type of the procedure was hemithyroidectomy in 86 (48%), total thyroidectomy in 51 (28%), isthmectomy in 4 (2%), and parathyroid procedure in 40 (22%) patients. The final pathological diagnosis was benign in 158 (87%) and malignant in 23 (13%) patients. Laryngoscopic examination revealed that 14 study patients had new unilateral postoperative VFP. Thirteen completed and returned the postoperative VHI questionnaire. Two weeks postoperatively, 11 of these patients and 20 control patients with no VFP returned for the voice recording.

**Fig. 1.**
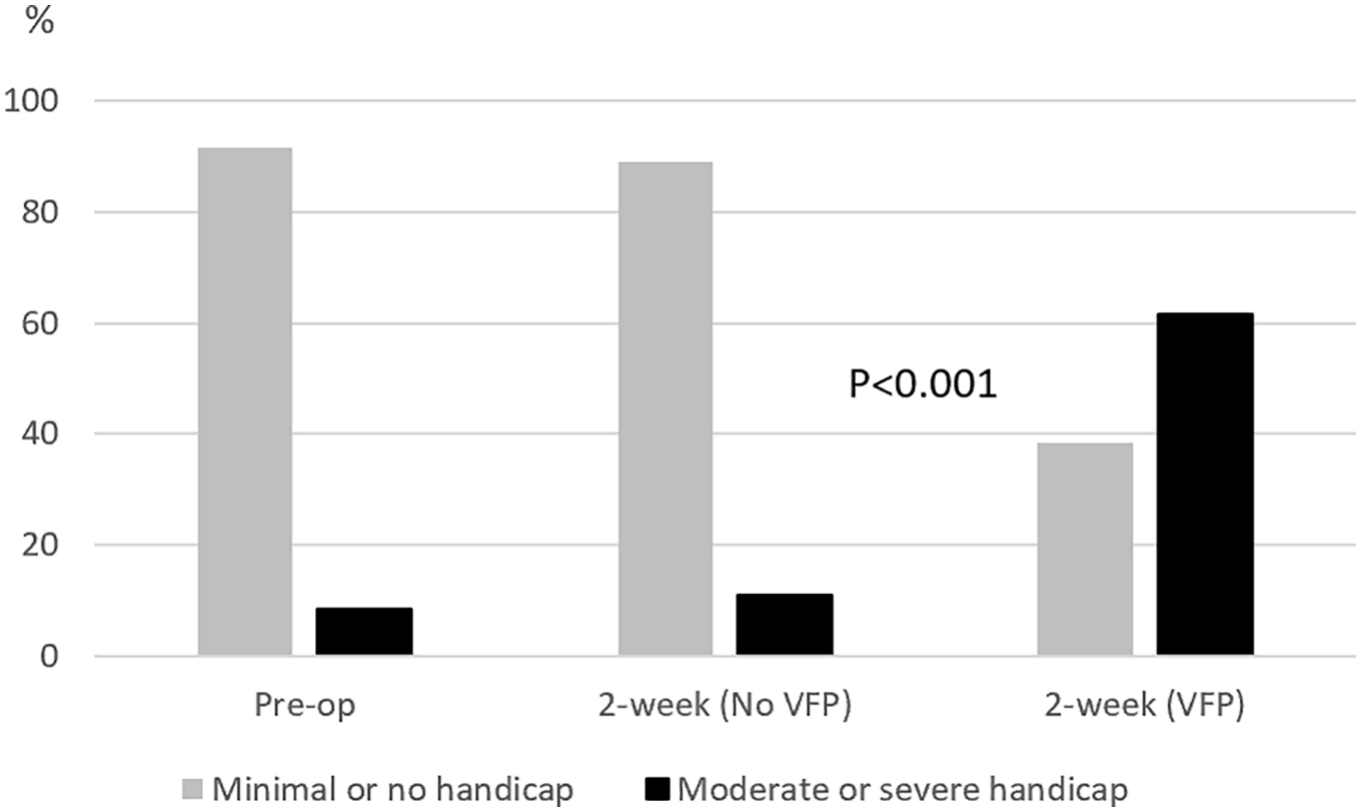
Distribution of classified Voice Handicap Index (VHI) scores preoperatively and 2 weeks after surgery. Postoperative scores are presented separately for patients with vocal fold paresis or paralysis (VFP) and without paresis or paralysis (No VFP). VHI scores 0–30 indicate minimal or no handicap and 31–120 indicate moderate or severe handicap. The p-value shows that the proportion of patients with moderate or severe handicap to patients with minimal or no handicap 2 weeks after surgery is significantly higher among patients with VFP than without VFP.

Patients with postoperative VFP had significantly higher total VHI scores (p = 0.040, Table 1). Their mean and median postoperative total VHI scores were 31 ± 31 and 32 (IQR, 3–50); for patients with no postoperative VFP, these were 11 ± 15 and 6 (IQR, 1–16). Two weeks after surgery, F-VHI and P-VHI subscales were significantly higher in patients with VFP (p = 0.007 and p = 0.006), with a statistically non-significant trend for higher E-VHI (p = 0.052). In addition, patients with postoperative VFP had a significantly greater increase between preoperative and postoperative total VHI scores, compared to patients without postoperative VFP (p = 0.028). Postoperatively, 2 (15%) patients with VFP reported severe handicap; 6 (46%) had moderate and 5 (39%) had minimal or no handicap. For patients without VFP, these numbers were 1 (1%), 16 (10%), and 138 (89%). The proportion of patients with moderate or severe handicap was significantly higher among patients with VFP than in those without (62% versus 11%, p < 0.001, Fig. 1).

**Table 1.** Median (M) and interquartile ranges (IQRs) of VHI total score and F-VHI, P-VHI, and E-VHI subscale scores preoperative and 2 weeks after surgery for patients with vocal fold paresis or paralysis (VFP) and without paresis or paralysis (No VFP); the median changes between the preoperative and 2-week values are shown in the right column.

	Pre-op			Post-op			Voice change		
	No VFP (n = 166)	VFP (n = 14)	p-value	No VFP (n = 154)	VFP (n = 13)	p-value	No VFP (n = 154)	VFP (n = 12)	p-value
VHI total	6.00 (2.00–13.72)	10.00 (3.50–27.00)	0.199	5.50 (1.00–16.25)	32.00 (3.00–50.00)	0.040^a^	0.00 (−4.00–3.00)	8.00 (0.00–28.00)	0.028^a^
F-VHI	2.00 (0.00–5.00)	3.00 (1.75–7.75)	0.106	1.00 (0.00–5.00)	11.00 (2.00–17.00)	0.007^a^	0.00 (−2.00–1.00)	5.00 (−1.00–11.00)	0.069
P-VHI	2.00 (0.00–6.00)	4.00 (0.00–11.50)	0.693	3.00 (0.00–8.00)	16.00 (1.50–22.50)	0.006^a^	0.00 (−2.00–2.00)	4.50 (1.00–10.75)	0.003^a^
E-VHI	0.00 (0.00–3.00)	2.50 (0.75–5.50)	0.046^a^	0.00 (0.00–4.00)	6.50 (0.00–12.75)	0.052	0.00 (−1.00–0.98)	0.50 (−1.75–9.75)	0.258

VHI: Voice Handicap Index; F/P/E-VHI: VHI scores describing functional/physical/emotional handicap.

a Statistically significant p-values.

**Fig. 2.**
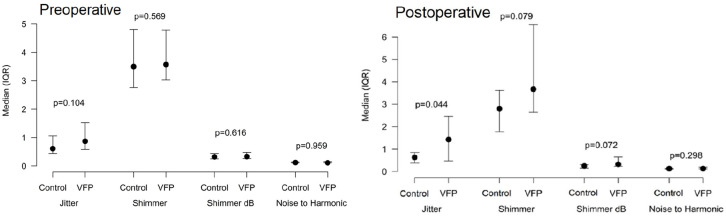
Median with interquartile ranges (IQRs) for Jitter, Shimmer, Shimmer dB and noise-to-harmonic ratio preoperative and 2 weeks after surgery for patients with vocal fold paresis or paralysis (VFP) and controls without paresis or paralysis.

Two weeks after surgery, in the objective voice analysis of the recorded samples, patients with postoperative VFP had significantly more jitter, compared to those without postoperative VFP (p = 0.044, Table 2). In their acoustic parameters recorded 2 weeks postoperatively, between patients with and without postoperative VFP, no other statistically significant difference emerged (Fig. 2). In this acoustic voice analysis, a comparison between patients with postoperative VFP preoperatively and 2 weeks postoperatively revealed no significant change in the parameters.

**Table 2.** Median (M) and interquartile ranges (IQRs) of F0, Jitter, Shimmer, Shimmer dB, and noise-to-harmonic ratio preoperative and 2 weeks after surgery for patients with vocal fold paresis or paralysis (VFP) and without paresis or paralysis (No VFP); the median changes between the preoperative and 2-week values are shown in the right column.

	Pre-op			2 weeks			Voice change		
	No VFP (n = 20)	VFP (n = 14)	p-value	No VFP (n = 20)	VFP (n = 11)	p-value	No VFP (n = 20)	VFP (n = 11)	p-value
F0	219.67 (196.21–253.14)	222.10 (170.59-258.98)	0.849	216.89 (184.20–241.43)	235.15 (194.39–253.07)	0.555	−9.34 (−26.06–18.42)	−5.08 (−23.26–9.55)	1.000
Jitter	0.61 (0.44–1.06)	0.87 (0.58–1.53)	0.104	0.63 (0.38–0.85)	1.43 (0.47–2.46)	0.044^a^	0.06 (−0.24–0.31)	0.11 (−0.21–0.96)	0.338
Shimmer	3.50 (2.76–4.81)	3.57 (3.03–4.79)	0.569	2.80 (1.77–3.62)	3.67 (2.65–6.55)	0.079	−1.02 (−2.07–0.25)	−0.25 (−1.10–1.43)	0.095
Shimmer dB	0.32 (0.25–0.44)	0.33 (0.26–0.48)	0.616	0.25 (0.15–0.31)	0.32 (0.23–0.65)	0.072	−0.10 (−0.19–0.13)	0.02 (−0.10–0.11)	0.066
Noise-to-harmonic ratio	0.12 (0.11–0.14)	0.11 (0.11–0.15)	0.959	0.13 (0.11–0.13)	0.13 (0.12–0.19)	0.298	0.00 (−0.01–0.02)	0.01 (−0.01–0.05)	0.427

a Statistically significant p-value.

Among the acoustic parameters of all patients 2 weeks after surgery, shimmer and shimmer dB had moderate to strong correlation with total VHI, F-VHI, P-VHI, and E-VHI scores; whereas NHR had moderate correlation with E-VHI (Table 3). However, when patients were considered in two groups—with and without postoperative VFP, no statistically significant correlation emerged.

**Table 3.** Pearson’s correlation coefficients representing the correlation between 2-week objective voice parameters (F0, Jitter, Shimmer, Shimmer dB, and noise-to-harmonic ratio) and VHI scores (total score and functional (F), physical (P), and emotional (E) subscale scores) for all patients.

	VHI total	F-VHI	P-VHI	E-VHI
F0	0.203	0.163	0.085	0.056
Jitter	0.291	0.302	0.231	0.312
Shimmer	0.467^a^	0.429^a^	0.394^a^	0.604^a^
Shimmer dB	0.542^b^	0.483^a^	0.458^a^	0.695^b^
Noise-to-harmonic ratio	0.308	0.279	0.287	0.457^a^

VHI: Voice Handicap Index.

a Correlation is significant at the 0.05 level (two-tailed).

b Correlation is significant at the 0.01 level (two -tailed).

Values between 0.1 and 0.3 represented mild correlation, from 0.3 to 0.5 moderate correlation, and more than 0.5. strong correlation.

Potential diagnostic tools based on ROC analyses are presented in Table 4. A total VHI score > 30 after surgery had 29% positive predictive value, 96% negative predictive value, 55% sensitivity, and 90% specificity, for VFP. A change between preoperative and postoperative (2 weeks after surgery) P-VHI scores > 0 had the best sensitivity, 83%, with 62% specificity, 15% positive predictive value, and 98% negative predictive value. Again, 2 weeks postoperatively, in MDVP analysis, jitter > 1.33 had the best specificity, 95%, with 55% sensitivity, 86% positive predictive value, and 79% negative predictive value. The best Youden index was achieved in postoperative P-VHI > 12 and postoperative jitter > 1.33, both 0.50. Combining two or more diagnostic tools failed to yield a better sensitivity, specificity, or Youden index.

**Table 4.** Assessment of potential diagnostic tools for vocal fold paresis or paralysis 2 weeks after thyroid or parathyroid surgery.

	AUC (95% confidence intervals)	p-value	Cut-off value	Sensitivity (%)	Specificity (%)	Positive predictive value (%)	Negative predictive value (%)	Youden’s index
2-week VHI total	0.684 (0.484, 0.884)	0.042	>30	55	90	29	96	0.45
F-VHI	0.735 (0.556, 0.914)	0.009	>10	55	90	29	97	0.45
P-VHI	0.727 (0.547, 0.906)	0.007	>12	62	88	31	97	0.50
E-VHI	0.654 (0.460, 0.848)	0.076	>4	58	79	18	96	0.37
Change VHI total	0.699 (0.520, 0.877)	0.028	>7	55	83	19	96	0.38
F-VHI	0.662 (0.455, 0.869)	0.074	>4	55	89	27	96	0.43
P-VHI	0.761 (0.606, 0.915)	0.003	>0	83	62	15	98	0.46
E-VHI	0.594 (0.379, 0.808)	0.281	>4	42	88	22	95	0.30
2-week jitter	0.723 (0.501, 0.944)	0.043	>1.333	55	95	86	79	0.50
Shimmer	0.695 (0.486, 0.905)	0.076	>4.765	46	95	83	76	0.41
Shimmer dB	0.700 (0.491, 0.909)	0.069	>0.424	46	95	83	76	0.41
NHR	0.618 (0.405, 0.831)	0.283	>0.187	27	100	100	71	0.27
Change jitter	0.609 (0.381, 0.838)	0.322	>0.689	36	95	80	73	0.31
Shimmer	0.686 (0.485, 0.888)	0.091	>–0.416	64	70	54	78	0.34
Simmer dB	0.705 (0.498, 0.911)	0.063	>–0.042	73	70	57	82	0.43
NHR	0.593 (0.361, 0.826)	0.397	>0.007	64	65	50	77	0.29

AUC: area under the curve; VHI: Voice Handicap Index; VHI total: VHI total score; F/P/E-VHI: VHI scores describing functional/physical/emotional handicap; NHR: noise-to-harmonic ratio.

In our validation of the recording technique, all the parameters in the recordings with the iPad and those recorded directly in the MDVP software showed strong correlation. The Pearson correlation coefficient was 0.95 for F0, 0.85 for jitter, 0.77 for shimmer, 0.84 for shimmer dB, and 0.75 for NHR.

## Discussion

This study demonstrated that, 2 weeks after surgery, patient self-assessment of voice using the VHI questionnaire as well as the jitter parameter in MDVP voice analysis show high specificity for postoperative VFP. The specificity for a total VHI score > 30 indicating moderate or severe handicap was 90%; this means that, in 10 patients with no postoperative VFP, only one had significant findings. The specificity for jitter > 1.33 was 95%; that is, only 5% patients with no VFP had a false positive finding related to this parameter and cut-off value. Moreover, jitter > 1.33 had a positive prediction value of 86%. Hence, jitter higher than this cut-off value at 2 weeks indicated a high probability of postoperative VFP. In contrast, the positive prediction value of a total VHI score > 30 was only 29%. Furthermore, both these parameters—jitter and total VHI—had poor sensitivity for postoperative VFP, only 55%. Thus, in MDVP voice or VHI analysis, nearly half of the patients with VFP had no significant findings. Therefore, these parameters are unsuitable for screening of postoperative VFP. The change between preoperative and postoperative P-VHI measurements had the best sensitivity (83%) for postoperative VFP, but the specificity was poor (63%).

The VHI questionnaire is useful when assessing patients’ voice problems. It represents patients’ voice-related quality of life, something that objective outcomes do not. In addition, patients’ self-assessment of their handicap allows us to identify patients who may benefit from speech therapy or surgical treatment of VFP. Furthermore, the VHI questionnaire is freely available, simple to fill in, and easy to interpret. Previously, the use of the VHI 2 weeks after thyroidectomy has proved an efficient tool to identify patients with voice problems early after surgery, with an 88% positive predictive value and 97% negative predictive value for voice dysfunction.^22^ That finding appears in a prospective observational longitudinal study comprising 91 patients with pre- and postoperative VHI scores, a study which compared these findings to those in videolaryngoscopy, blinded assessment of voice by experienced clinicians, acoustic recordings, and aerodynamic assessments. In that study, voice dysfunction was defined according to laryngoscopic examination, acoustic, auditory perceptual, and patient report parameters.

Many previous studies suggest that the VHI questionnaire may be useful to identify patients with postoperative VFP after thyroid surgery. In a prospective cohort study comprising 62 patients undergoing surgery for benign nodular thyroid disease, Sorensen et al.^15^ showed significantly higher (p = 0.002) postoperative VHI scores among patients with paresis than among those without; they specifically examined paresis of the recurrent laryngeal nerve or the external branch of the superior laryngeal nerve. In another study, 110 thyroid carcinoma patients with no postoperative VFP used a VHI-10 questionnaire and had no change in preoperative–postoperative VHI scores.^23^ The VHI-10 is a validated and abbreviated version of the original VHI.^24^ It has 10 questions and the total score ranges from 0 to 40. A cross-sectional study of 2325 patients with thyroid carcinoma who were surgically treated showed that patients with postoperative VFP were affected by a voice handicap that could be detected by means of a VHI-10 questionnaire 2 to 4 years after surgery.^16^ That study found 61% patients who reported postoperative VFP to have a VHI-10 score higher than 11, compared to 10% of patients without VFP (p < 0.001).

In this study, patients with postoperative VFP had significantly higher total VHI scores than patients without postoperative VFP did. This is consistent with the study by Sorensen et al.^15^ Moreover, in a recent prospective multicenter study consisting of 800 patients with benign or non-extensive malignant thyroid diseases undergoing total thyroidectomy, Borel et al.^17^ demonstrated that median VHI scores were significantly higher in patients with postoperative VFP 2 months after surgery. The median total VHI score was 14 (IQR 3–39) in patients with postoperative VFP compared to 4 (IQR 0–20) in those without (p = 0.004). The results of this study are in line with the study by Borel and colleagues, although patients with postoperative VFP had higher median scores 32 (IQR 3–50) versus 6 (IQR 1–16) (p = 0.040) in those without postoperative VFP. The reason for the higher scores may be that the patients completed the VHI questionnaire earlier in our study, 2 weeks after surgery, without potential recovery from the paresis or paralysis or the development of compensatory mechanisms.

This study identified a VHI total score of 30 as a cut-off to identify patients with postoperative VHI, with 55% sensitivity and 90% specificity. In a study of 355 patients, Van Gogh et al.^25^ compared groups of individuals with voice disorders and those with normal voice. They determined that a cut-off value of 15 in the total VHI score may identify patients with voice problems in their daily life (sensitivity 97%, specificity 86%). The higher cut-off value in our study may indicate that, after thyroid or parathyroid surgery, factors other than postoperative VFP may cause voice disorders, such as intubation trauma, laryngeal edema, swelling of the recurrent laryngeal nerve, strap muscles division or injury, laryngotracheal fixation, and postoperative pain.^17^ A VHI score higher than 15 may be necessary to differentiate VFP from other voice disorders.

In this study, ROC analysis demonstrated that, with the total VHI score more than seven points higher postoperatively than preoperatively, the specificity for VFP was fairly good at 83%, but the negative prediction value was only 19%. The original article of VHI development and validation, a study of 63 patients, stated that a shift ⩾ 18 points in the total score is required to rule out change due to normal variation.^13^ However, some VHI studies have lower cut-offs. Two studies compared patients with voice problems to healthy controls. One study with 355 patients suggested a cut-off value of 10 for VHI change; the other, with 72 patients, determined an increment of total VHI score by 13 as indicating clinically relevant deterioration of voice.^25,26^

To our knowledge, no previous studies have investigated VHI as a screening method for VFP after thyroid or parathyroid surgery. However, Nam et al.^27^ developed their own voice questionnaire, which contained questions from the VHI along with other questions describing subjective symptoms related to thyroid surgery. Their questionnaire consisted of 20 questions scored on a 5-point progressive numeric scale similar to the VHI. Based on this new questionnaire, they developed a voice-screening protocol for thyroid surgery and piloted it with 242 patients who underwent thyroidectomy for benign or malignant thyroid tumors.^28^ Patients completed the questionnaire 2 weeks after surgery. A score of 25 or more was the cut-off value to identify patients with postoperative VHI. For postoperative VFP, the sensitivity was 100%, specificity 50%, positive predictive value 8%, and negative predictive value 100%. Compared to the results of our study, Nam and colleagues’ thyroidectomy-related voice questionnaire had excellent sensitivity and no missed patients with postoperative VFP. Their specificity was poor, however; half of the patients with no postoperative VFP still needed laryngoscopic examination after a questionnaire screening. According to our study, VHI is not an ideal screening test for VFP after thyroid or parathyroid surgery; neither is the thyroidectomy-related voice questionnaire by Nam and colleagues.

In this study, postoperatively, jitter was significantly greater among patients with VFP compared to patients with no VFP. However, we found no significant change between pre- and postoperative acoustic voice analysis measures in patients with postoperative VFP. In contrast is a prospective study by Chun et al.^29^ They used MDVP analysis of 300 patients preoperatively and 2 weeks after thyroidectomy surgery to demonstrate that postoperative jitter, shimmer, and NHR were significantly greater than preoperative values among 31 patients with VFP.^29^ The insignificant values in this study may be due to the low number of patients with VFP.

### Study limitations

The low number of patients with postoperative VFP in this study may underestimate the value of the screening tests because of the possibility of type 2 statistical error. We did not record voice samples directly in the MDVP software. Instead, for logistic reasons, we recorded using an iPad; we then transferred the samples to an MDVP workstation at a different location. This may have caused changes in sound quality. However, this method was validated in 31 patients and proved to be highly accurate compared to recording directly in the MDVP workstation with a standard microphone.

## Conclusion

Two weeks after thyroid or parathyroid surgery, both patient self-assessment with the VHI and jitter in acoustic voice analysis have a high specificity but poor sensitivity for VFP. Without routine laryngoscopy, approximately half of the patients with postoperative VFP could be overlooked. However, 2 weeks postoperatively, if patients have no voice disturbance complaints, the likelihood of VFP is low. Further studies are needed to create an accurate screening test for postoperative VFP.

## Supplemental Material

sj-pdf-1-sjs-10.1177_14574969211007036 – Supplemental material for Patient Self-Assessment and Acoustic Voice Analysis in Screening of Postoperative Vocal Fold Paresis and Paralysis Click here for additional data file. Supplemental material, sj-pdf-1-sjs-10.1177_14574969211007036 for Patient Self-Assessment and Acoustic Voice Analysis in Screening of Postoperative Vocal Fold Paresis and Paralysis by Maria Heikkinen, Elina Penttilä, Mari Qvarnström, Kimmo Mäkinen, Heikki Löppönen and Jussi M Kärkkäinen in Scandinavian Journal of Surgery
