# Supplementary figures and images for: Patient Self-Assessment and Acoustic Voice Analysis in Screening of Postoperative Vocal Fold Paresis and Paralysis
Source: Scand J Surg. 2021 Apr 12;110(4):524–32. doi: 10.1177/14574969211007036 (PMC8688980; doi:10.1177/14574969211007036)

## Study Flowchart

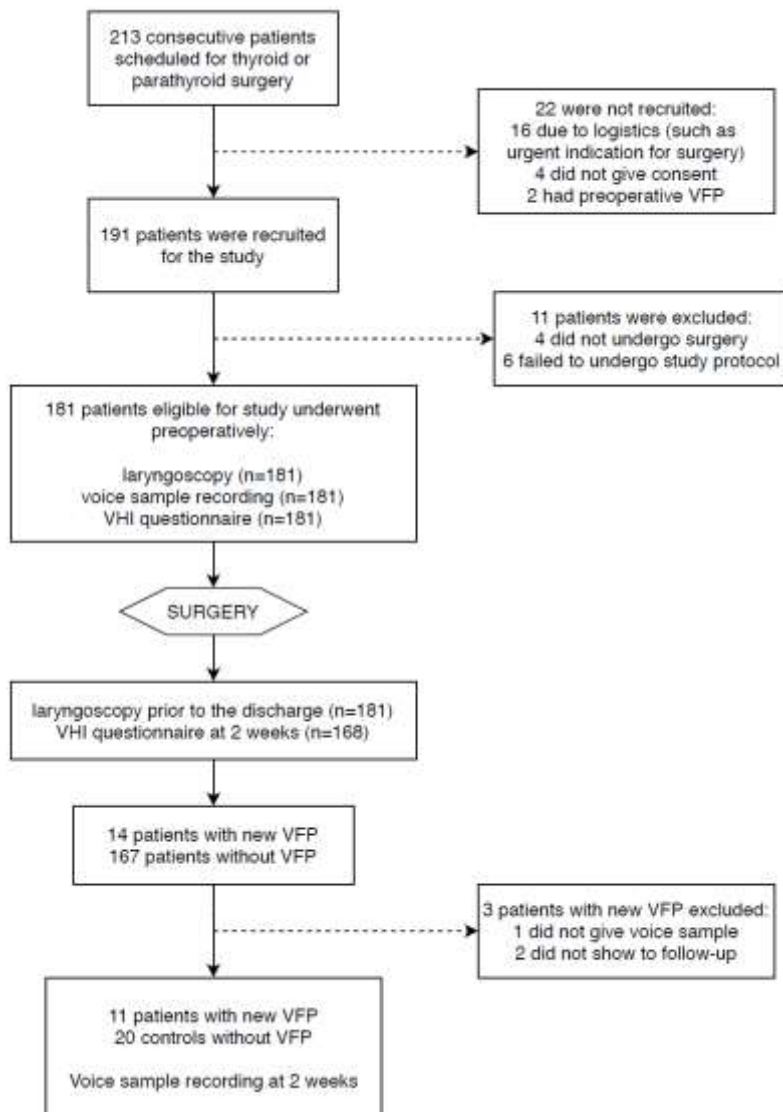

VFP vocal fold paresis or paralysis, VHI Voice handicap index

Supplement: sj-pdf-1-sjs-10.1177_14574969211007036 – Supplemental material for Patient Self-Assessment and Acoustic Voice Analysis in Screening of Postoperative Vocal Fold Paresis and Paralysis [file sj-pdf-1-sjs-10.1177_14574969211007036.pdf]
